# Determining Physiological and Psychological Predictors of Time to Task Failure on a Virtual Reality Sørensen Test in Participants With and Without Recurrent Low Back Pain: Exploratory Study

**DOI:** 10.2196/10522

**Published:** 2018-09-10

**Authors:** Megan E Applegate, Christopher R France, David W Russ, Samuel T Leitkam, James S Thomas

**Affiliations:** 1 Wyss Institute for Biologically Inspired Engineering Cambridge, MA United States; 2 Department of Psychology Ohio University Athens, OH United States; 3 Department of Physical Therapy Virginia Commonwealth University Richmond, VA United States; 4 Department of Physical Medicine and Rehabilitation Virginia Commonwealth University Richmond, VA United States; 5 School of Physical & Occupational Therapy McGill University Montreal, QC Canada

**Keywords:** fatigue, low back pain, Sørensen test, trunk mass, virtual reality

## Abstract

**Background:**

Sørensen trunk extension endurance test performance predicts the development of low back pain and is a strong discriminator of those with and without low back pain. Performance may greatly depend on psychological factors, such as kinesiophobia, self-efficacy, and motivation. Virtual reality video games have been used in people with low back pain to encourage physical activity that would otherwise be avoided out of fear of pain or harm. Accordingly, we developed a virtual reality video game to assess the influence of immersive gaming on the Sørensen test performance.

**Objective:**

The objective of our study was to determine the physiological and psychological predictors of time to task failure (TTF) on a virtual reality Sørensen test in participants with and without a history of recurrent low back pain.

**Methods:**

We recruited 24 individuals with a history of recurrent low back pain and 24 sex-, age-, and body mass index–matched individuals without a history of low back pain. Participants completed a series of psychological measures, including the Center for Epidemiological Studies-Depression Scale, Pain Resilience Scale, Pain Catastrophizing Scale, Tampa Scale for Kinesiophobia, and a self-efficacy measure. The maximal isometric strength of trunk and hip extensors and TTF on a virtual reality Sørensen test were measured. Electromyography of the erector spinae, gluteus maximus, and biceps femoris was recorded during the strength and endurance trials.

**Results:**

A two-way analysis of variance revealed no significant difference in TTF between groups (*P*=.99), but there was a trend for longer TTF in females on the virtual reality Sørensen test (*P*=.06). Linear regression analyses were performed to determine predictors of TTF in each group. In healthy participants, the normalized median power frequency slope of erector spinae (beta=.450, *P*=.01), biceps femoris (beta=.400, *P*=.01), and trunk mass (beta=−.32, *P*=.02) predicted TTF. In participants with recurrent low back pain, trunk mass (beta=−.67, *P*<.001), Tampa Scale for Kinesiophobia (beta=−.43, *P*=.01), and self-efficacy (beta=.35, *P*=.03) predicted TTF.

**Conclusions:**

Trunk mass appears to be a consistent predictor of performance. Kinesiophobia appears to negatively influence TTF for those with a history of recurrent low back pain, but does not influence healthy individuals. Self-efficacy is associated with better performance in individuals with a history of recurrent low back pain, whereas a less steep median power frequency slope of the trunk and hip extensors is associated with better performance in individuals without a history of low back pain.

## Introduction

Low back pain (LBP) represents a significant societal and economic burden [1] with direct medical costs approaching US $100 billion annually in the United States alone [2]. These costs are driven primarily by the 10%-15% of individuals who develop chronic LBP [1,3]. Poor trunk extension endurance has been identified as a risk factor for the development of LBP [4]. Specifically, poor time to task failure (TTF) on the Sørensen back extension endurance test, which requires an individual to maintain the upper body in an unsupported horizontal position to the point of fatigue, predicts first-time episodes of LBP [5,6] and chronic LBP [7]. Although the Sørensen test has been used for the identification of LBP risk, the underlying mechanisms driving poor performance on the test are not well understood. Research has demonstrated that in addition to physiological factors, such as median power frequency (MPF) slopes of the trunk and hip extensors and anthropometrics, psychological factors must be considered in the assessment of performance on the Sørensen test.

Evidence suggests that individuals terminate the Sørensen test for reasons other than subjective fatigue; these reasons include pain, discomfort, fear, and lack of motivation [8]. Studies of rehabilitation motivation in clinical populations, such as those of individuals recovering from cardiac events, have demonstrated that although motivation is difficult to define and measure, it plays a crucial role in rehabilitation outcomes for patients [9]. Motivation could be manipulated through the use of distracting, immersive virtual reality gaming. Virtual reality games have been implemented for pain distraction in individuals receiving chemotherapy and during burn debridement, resulting in lower pain ratings and greater tolerance of treatments [10-12] and encouraging greater lumbar spine flexion in individuals with kinesiophobia and LBP [13]. Thus, by providing a distraction element to counteract fear cognitions during the assessment, a virtual reality video game could enhance motivation during the Sørensen test, leading to maximal effort.

However, psychological factors are likely still involved in performance on the Sørensen test. Self-efficacy contributes to the performance of physical activities. Self-efficacy is the magnitude of belief in one’s ability to perform a certain task to achieve a specific outcome [14]. Self-efficacy is strongly associated with sports performance [15]. Although the study of self-efficacy regarding physical activity contributes to the understanding of human behavior, application to the Sørensen test, specifically, is limited.

The influence of fear of pain may vary as a function of prior LBP experience. Chronic pain is a biopsychosocial phenomenon; an individual’s emotions and appraisal of pain contributes to chronicity [16]. Cognitive appraisal of pain varies based on individuals’ beliefs about their ability to cope with pain. In many situations, pain can elicit negative emotional reactions that lead to the amplification of pain experiences [17]. Individuals with maladaptive emotional responses to pain who undergo traditional treatment for LBP may continue to experience pain and disability long after the symptoms are treated. A cognitive behavioral model of chronic LBP, termed the fear-avoidance model, explains the progression from acute pain to chronic pain and disability [18,19]. In addition, the model hypothesizes that an individual’s pain experience depends upon their established levels of pain-related fear. Kinesiophobic individuals, those who are prone to avoidance of movement for fear of pain or harm, respond to pain with catastrophic thoughts (ie, “The pain will get worse if I attempt to overcome it”), leading to inactivity and further progression of disability [18,20].

Measures of kinesiophobia have been used to predict LBP [19]. Pain-related fear predicts reduced maximal force production and increased pain-related interference in daily activities, regardless of actual pain levels [21]. Moreover, kinesiophobia has been recognized as an integral factor in Sørensen test performance. Sørensen TTF “underperformance” in individuals with chronic LBP could be predicted, in part, by fear-avoidance beliefs, as well as self-efficacy [22].

A variation of the Sørensen test that uses a virtual reality video game could encourage maximal effort, counteract fear cognitions, and allow for more accurate identification of both the physiological and psychological factors driving performance. Accordingly, we have developed a variation of the Sørensen test that uses a virtual reality video game to provide motivation and distraction. This study aims to determine whether the use of a virtual reality video game influences performance on the Sørensen test and whether the predictors of TTF vary between individuals with and without recurrent LBP on the virtual reality Sørensen test.

## Methods

### Participants

A sample of 24 individuals (12/24, 50% male) with a history of recurrent LBP (LBP) and 24 individuals (12/24, 50% male) with no history of LBP (Healthy) matched for age, sex, and body mass index (BMI) were recruited from the Ohio University student population and surrounding community for this comparative study. Table 1 summarizes the participants’ characteristics. Individuals with a history of hip arthroscopy or spine surgery, known neurological, visual, or orthopedic impairments, depression, ongoing drug or alcohol problems, elevated resting blood pressure (>135/>90 mmHG), or BMI of >35 were excluded from the study. We defined LBP history as having experienced more than one episode of LBP with symptoms occurring in the past 6 months and a previous consultation regarding their LBP symptoms with a health care provider; participants reporting moderate to severe pain (numerical pain rating scale of >3) within the past 6 weeks or those who did not meet the classification of category 1 (LBP that does not radiate) through category 3 (LBP that radiates beyond the knee but without neurological signs) on the Classification System of the Quebec Task Force on Spinal Disorders were excluded from participation. The protocol was approved by the Ohio University Institutional Review Board for human subjects research, and all individuals provided written consent prior to participation.

### Instruments

#### Center for Epidemiological Studies-Depression Scale

The Center for Epidemiological Studies-Depression Scale (CES-D) was used in the general population to measure depressive symptomatology. Good predictive validity for the identification of depression in individuals with chronic pain has been established [22] as well as good sensitivity (93.2%) using a cutoff score of 19 (out of 60) for the identification of depression in individuals with chronic pain [23].

#### Pain Catastrophizing Scale

The Pain Catastrophizing Scale (PCS) is a 13-item scale that measures pain catastrophizing by assessing the degree to which the respondent experiences specific thoughts and feelings during pain on a 5-point Likert-type scale with the endpoints “Not at all” (0) and “All the time” (4) with a score of 30 indicating clinically relevant pain catastrophizing behavior. PCS has been identified as a reliable and valid measure of pain catastrophizing (Cronbach alpha=.87; test-retest intraclass correlation coefficient=.93). PCS is consistently associated with pain sensitivity and pain-related distress in experimental pain studies [24-26]. Furthermore, pain catastrophizing is a primary vulnerability construct [27,28].

#### Pain Resilience Scale

The Pain Resilience Scale (PRS) asks participants how they respond when faced with intense or prolonged pain by rating items on a 14-item Likert scale using a “Not at all” (0) to “All the time” (4) scale with higher scores indicating greater pain resilience. Strong internal consistency and acceptable levels of stability have been established (alpha=.93, intraclass correlation coefficient=.80) [29].

#### Tampa Scale for Kinesiophobia and Tampa Scale for Kinesiophobia-General Population

Two versions of the Tampa Scale for Kinesiophobia (TSK) were used in this study, each using 17 items on a 4-point Likert scale ranging from “Strongly disagree” (1) to “Strongly agree” (4) with scores >36 indicating clinically relevant kinesiophobia. The LBP group completed the standard TSK, which assessed fear of movement at the risk of injury or (re)injury. The healthy group completed the TSK-General Population (TSK-G), which used items modified to ask how much the respondent would fear movement at the risk of injury or (re)injury if they had LBP. In addition, construct validity and predictive ability has been established in LBP populations [30]. In the general population, TSK-G is also reliable and valid as a self-report measure of fear of movement and (re)injury [31].

#### Self-Efficacy Measure

The self-efficacy measure was developed for this study based on prior studies [32,33]. After practicing the task position for a brief period, participants were asked to indicate their confidence in their ability to maintain the Sørensen test position for 1, 2, 3, and 4 minutes on a scale ranging from “Not at all confident” (0) to “Highly confident” (100).

### Data Collection

Participants completed 2 separate testing sessions. Participants in this study were first included in an assessment of performance on the classic Sørensen test [34] and were invited to participate in the virtual reality Sørensen test 3-14 days later. During the first testing session, participants completed the psychological surveys, maximal strength assessments, and the classic Sørensen test. During the second testing session, participants completed the virtual reality Sørensen test.

#### Electromyography Data

Electromyography (EMG) was performed as described previously [34]. In brief, EMG was collected using a 16-channel Delsys Bagnoli system (Delsys Inc, Boston, MA, USA; bandwidth 20-450 Hz); the bar leads were modified with clip leads to allow attachment to Ag-Ag Cl surface electrodes over the erector spinae (ERS) at the L2 and L4 level aligned between the posterior superior iliac spine and the lateral border of the muscle at the 12th rib, gluteus maximus midway between the greater trochanter and the posterior superior iliac spine, and long head of the biceps femoris (BF) midway between the fibular head and the ischial tuberosity. The raw surface EMG data were amplified (1k) and A/D converted with 16-bit resolution, sampled at 1000 Hz, and averaged across sides for each muscle.

#### Median Power Frequency

MPF was calculated as described previously [35]. Using a fast Fourier transformation with a 512-point Hamming window, the EMG power spectrum for each muscle was calculated. MPF was determined using a 2-second moving window with 50% overlap. The normalized slope of MPF was determined as follows: (MPF slope/initial MPF)x100 [35]. All processing of EMG data were performed with custom software written in MATLAB (Version 2016b; The MathWorks).

**Table 1:** Participant characteristics.

Characteristics	Healthy (n=24), mean (SE)	Low back pain (n=24), mean (SE)
Age (years)	29.2 (2.2)	24.3 (1.5)
Height (m)	1.7 (0.0)	1.7 (0.0)
Weight (kg)	73.3 (2.6)	71.4 (2.6)
Body mass index (kg/m^2^)	24.8 (0.7)	24.2 (0.7)

#### Force Output and Torque Moment Data

The maximal voluntary contraction data were measured as described previously [34]. In brief, our custom articulated fatigue table integrated a 6 degree of freedom (DOF) load cell (MC5-1250; AMTI, Watertown, MA, USA) into the trunk platform connected to a signal conditioner (GEN 5; AMTI), and single DOF load cell (XTS4-500; Load Cell Central, Milan, PA, USA) into the leg brace connected to an analog signal conditioner (OM19; Load Cell Central). Force and torque data were A/D converted at 16-bit resolution and sampled at 1000 Hz.

#### Position Data

The trunk position during the Sørensen test was measured as described previously [34]. In brief, custom-made potentiometers were anchored over the participant’s sacrum at the level of L5-S1 and trunk at the level of T12-L1. An algorithm converted the potentiometers’ voltages into position (degrees of rotation) with excellent linearity of fit (*R*^2^=0.9988). Our custom LABVIEW (Version 13; National Instruments, Austin, TX, USA) program used the algorithm to track the position during the Sørensen tests. The horizontal position was individually calibrated prior to each test.

### Maximal Voluntary Contraction Procedure

**Figure 1:**
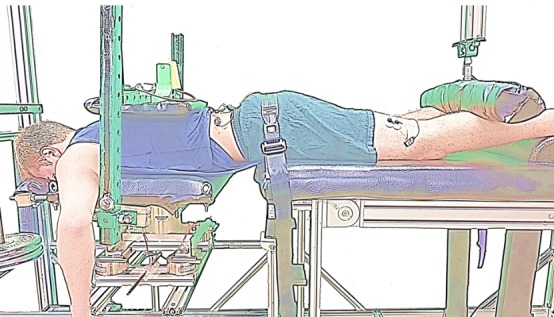
Experimental setup on the fatigue table for maximal voluntary contraction of the trunk and hip.

The maximal voluntary contraction procedure was completed as described previously [34]. As illustrated in Figure 1, subjects were situated on the custom fatigue table with the anterior superior iliac spine aligned with the edge of the table and the torso supported by the platform positioned such that the trunk center of mass was centered over the 6 DOF load cell. In addition, the torso was secured to the platform, the pelvis was secured to the table, and the lower legs were secured at 33% of hip height by a padded bar connected to the single DOF load cell. Bracing of the feet was inhibited with a foam roll placed below the ankles. The trunk mass was measured in this position. EMG was measured as previously described and the custom LABVIEW (Version 13; National Instruments) program collected EMG and load cell measurements.

For the trunk extension trials, participants were instructed to pull their torso up into the back restraint. Three submaximal trunk extension attempts of increasing intensity were followed by 3 maximal trunk extension attempts. Then, participants were instructed to extend their legs up against the stationary leg restraint; 3 submaximal hip extension attempts of increasing intensity were followed by 3 maximal attempts. We provided 2 minutes of rest between each attempt. Verbal encouragement and visual and audio feedback were provided via the custom LABVIEW (Version 13; National Instruments) program.

### Virtual Reality Sørensen Procedure

**Figure 2:**
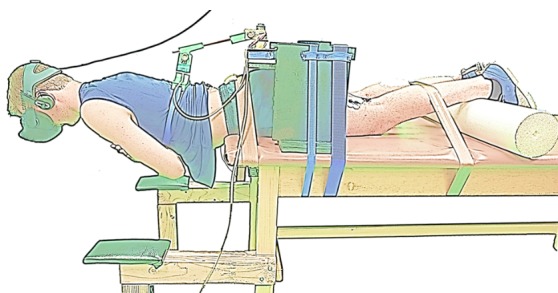
Experimental setup on the standard table for the virtual reality Sørensen test.

**Figure 3:**
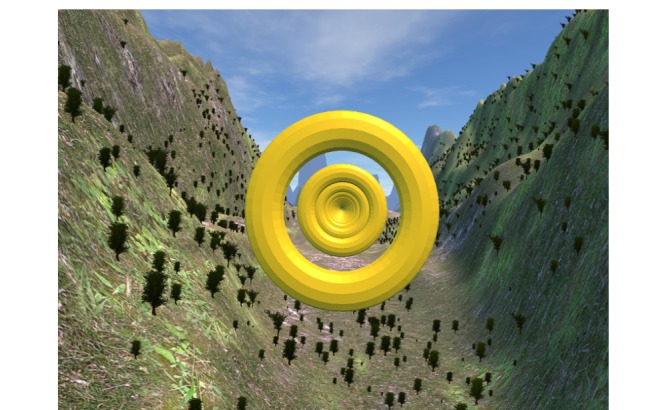
Participant’s perspective during gameplay, displayed through the Oculus Rift headset.

The participants performed the virtual reality Sørensen test on a standard table with belts across the pelvis and calves at 33% of hip height, the anterior superior iliac spine aligned with the edge of the table, and the upper body unsupported, as seen in Figure 2. Subjects wore an Oculus Rift head mounted display (Oculus Rift Developers Kit 2), as shown in Figure 2. During the test, the Oculus Rift displayed a sky environment in which the participant attempted to “fly” through hoops, as seen in Figure 3. Extending and flexing the trunk appeared to make the subject fly higher and lower, respectively. The hoops were positioned such that the participants were encouraged to maintain a horizontal position for as long as possible. Immediately following a brief practice attempt of <5 seconds, participants completed the self-efficacy questionnaire. 

Participants then attempted to maintain the task position until failure while receiving audio and visual feedback through the virtual reality video game; a tone played when the participant’s position was >2° beyond the target position in either direction, which was visually represented by flying above or below the hoops. The trial was terminated when participants fell out of the range (±2°) for >3 consecutive seconds.

### Statistical Analysis

In this study, independent-sample *t* tests were used to evaluate differences in participant demographics. A two-way analysis of variance was used to determine group and sex differences in TTF on the virtual reality Sørensen test. In addition, stepwise linear regression analyses were performed to determine which physiological factors were related to TTF on the virtual reality Sørensen test in each group. The second set of linear regression analyses was performed with the significant physiological factors entered into the first block and psychological factors entered stepwise in the second block to determine which psychological factors were related to TTF on the virtual reality Sørensen test in each group. All analyses were performed in SPSS (IBM Corp.), and results are reported as mean (SE) unless otherwise stated.

## Results

### Demographics

The independent-sample *t* test revealed no significant differences between Healthy and LBP groups with one exception; the depression scores were significantly higher in the LBP group than in those the healthy group (Table 2).

### Time to Task Failure

A 2 Group (Healthy, LBP) x 2 Sex (Male, Female) two-way analysis of variance revealed no significant differences in group (*F*_1,44_=0.00, *P*=.99) or group by sex (*F*_1,44_=0.33, *P*=.57; Table 3); however, there was a marginal effect of sex (*F*_1,44_=3.89, *P*=.06), which reflected a tendency toward the longer TTF in female versus male participants.

### Predictors of Virtual Reality Sørensen Time to Task Failure

Simple correlations were run between TTF and each of the physiological and psychological factors that were entered into the linear regression analyses. Table 4 displays the Pearson correlations.

#### Healthy

A stepwise linear regression analysis identified the normalized MPF slope of ERS (beta=.45, *P*=.01), normalized MPF slope of BF (beta=.40, *P*=.01), and trunk mass (beta=−.32, *P*=.03) as significant predictors of TTF. A separate linear regression analysis was then run with the MPF slopes of ERS and BF; trunk mass was entered into the first block and all psychological measures (ie, CES-D, TSK, self-efficacy, PCS, and PRS) were offered stepwise into the second block. Only trunk mass and the normalized MPF slopes of ERS and BF were retained as significant predictors of TTF on the virtual reality Sørensen test in the healthy group (Table 5).

#### Low Back Pain

A stepwise linear regression analysis identified the trunk mass (beta=−.53, *P*=.01) as a significant predictor of TTF. A separate linear regression analysis was then run with trunk mass entered into the first block, and all psychological measures (ie, CES-D, TSK, self-efficacy, PCS, and PRS) were offered stepwise into the second block. In the final model, the trunk mass (beta=−.67, *P*=.001), TSK (beta=−.43, *P*=.01), and self-efficacy (beta=.35, *P*=.03) were retained as significant predictors of TTF on the virtual reality Sørensen test in the LBP group (Table 5).

**Table 2:** Anthropometric, strength, and psychological survey measures.

Survey measures	Healthy (n=24), mean (SE)	Low back pain (n=24), mean (SE)	*P* value
Trunk mass (kg)	36.7 (1.5)	35.4 (1.6)	.54
Trunk length (m)	0.5 (0.0)	0.4 (0.0)	.15
Vertical trunk force (N)	476.7 (52.8)	511.7 (35.4)	.65
Trunk moment (Nm)	42.3 (4.1)	45.4 (5.4)	.66
Hip force (N)	131.0 (8.2)	138.4 (8.6)	.50
Erector spinae MPF^a^ slope (%/s)	−0.1 (0.0)	−0.1 (0.0)	.32
Gluteus maximus MPF slope (%/s)	−0.3 (0.0)	−0.2 (0.0)	.16
Biceps femoris MPF slope (%/s)	3.8 (0.8)	7.6 (1.2)	.38
Depression (0-60)^b^	39.3 (2.1)	37.7 (2.1)	.01
Pain resilience (0-56)^b^	7.6 (1.3)	6.8 (0.9)	.61
Pain catastrophizing (0-52)^b^	30.9 (1.2)	32.0 (1.3)	.59
Kinesiophobia (17-68)^b^	38.7 (4.1)	44.5 (3.2)	.54

^a^MPF: median power frequency.

^b^Ranges for the scales.

**Table 3:** Time to task failure on the virtual reality Sørensen test.

Participants	Healthy, mean (SE)	Low back pain, mean (SE)	Total, mean (SE)
Male	107.2 (9.6)	98.1 (9.7)	102.7 (6.7)
Female	128.7 (21.1)	137.3 (17.8)	133.0 (13.5)
Total	118.0 (11.6)	117.7 (10.7)	117.8 (7.8)

**Table 4:** Simple correlations between time to task failure and factors entered into the linear regression analyses.

Measures	Healthy		Low back pain	
	*r*	*P* value	*r*	*P* value
Trunk mass	−0.467	.02	−0.532	.01
Trunk length	0.180	.40	0.016	.94
Vertical trunk force	0.065	.76	−0.061	.78
Trunk moment	−0.248	.24	−0.200	.35
Hip force	−0.056	.80	−0.299	.16
Erector spinae MPF^a^ slope	0.616	.001	0.314	.14
Gluteus maximus MPF slope	0.440	.03	−0.174	.42
Biceps femoris MPF slope	0.513	.01	0.418	.04
Self-efficacy	0.525	.01	0.235	.27
Depression	0.128	.55	−0.194	.36
Pain catastrophizing	−0.158	.46	−0.091	.67
Pain resilience	−0.030	.89	−0.362	.08
Kinesiophobia	−0.106	.62	−0.276	.19

^a^MPF: median power frequency.

**Table 5:** Significant factors identified by the linear regression analyses.

Factor		Unstandardized beta	SE	Standardized beta	*t*	*P* value
**Healthy**						
	(Constant)	301.32	10.20	—	7.50	<.001
	Erector spinae MPF^a^	140.21	45.17	.45	3.10	.01
	Biceps femoris MPF	130.24	44.74	.40	2.91	.01
	Trunk mass	−1.12	0.49	−.32	−2.30	.03
**Low back pain**						
	(Constant)	342.21	61.68	—	5.55	<.001
	Trunk mass	−2.08	0.48	−.67	−4.34	<.001
	Kinesiophobia	−3.56	1.27	−.43	−2.81	.01
	Self-efficacy	1.15	0.49	.35	2.34	.03

^a^MPF: median power frequency.

## Discussion

### Principal Findings

This study aimed to examine the performance on a variation of the Sørensen test using a virtual reality video game in individuals with and without a history of recurrent LBP. To the best of our knowledge, this is the first study to use a virtual reality video game in conjunction with the Sørensen test. Contrary to much of the published literature, we did not find a significant difference in TTF between the groups. In the first longitudinal study, males with a short TTF on the Sørensen test were most likely to experience LBP in the following year, identifying the test as a predictor of first-time LBP [36]. The test was later recognized as a discriminator of those with and without LBP; individuals who had no prior LBP experience exhibited a markedly longer TTF than those with any LBP experience [37]. Many studies have reported consistent findings; however, others have failed to find a difference in performance between those with and without LBP. Many physiological factors, including the BMI, trunk mass, MPF slopes of the trunk and hip extensors, and maximal trunk and hip strength, have been found to influence the performance on the task; these factors have been discussed previously [34].

Although our sample of individuals with recurrent LBP performed just as well on the virtual reality Sørensen test as those without LBP, several interesting findings regarding the factors associated with TTF emerged. In the healthy group, it appears that TTF was driven primarily by trunk mass and the MPF slopes of the trunk and hip extensors, and this is consistent with our previous work [34]. In addition, trunk mass was a predictor of TTF in the LBP group. Other studies also demonstrated the marked effects of anthropometrics on performance. The workload of the task is governed by the weight of the body above the hips. It is obvious that an individual with a heavier trunk mass will not be able to maintain the test position for as long as another individual with the same strength capacity but lighter trunk mass. The effects of anthropometrics tend to be consistent in both individuals with and without LBP. Previously, marked associations have been identified between body mass, BMI, and the MPF slope of ERS in males and females with and without LBP [38]. Moreover, a marked association between TTF and torso mass in females with and without LBP has been demonstrated [39]. Trunk mass is an important factor in Sørensen test TTF, especially in those with a history of LBP, and should be considered when assessing performance. A variation in the Sørensen test that normalizes the workload to a consistent percentage of maximal strength would account for differences in trunk mass and strength to allow for a more objective assessment of endurance.

In this study, self-efficacy emerged as an important factor in Sørensen test performance. Motivation has long been recognized as a consequential factor in Sørensen test performance [22,36,40-43]; however, to the best of our knowledge, we are the first to attempt to manipulate it through the use of a virtual reality video game. The self-efficacy measure was created specifically for the Sørensen test task, which likely explains its strong association with TTF in the LBP group. Interestingly, self-efficacy was not predictive of performance in the healthy group. Thus, in this sample of individuals without a history of LBP, it appears that self-efficacy did not drive performance. Alternatively, our sample of individuals with a history of recurrent LBP performed better on the virtual reality Sørensen test if they reported higher ratings of confidence in their capacity to perform the task; this is consistent with our previous findings on the standard Sørensen test [34] as well as those obtained by others who found that performance was predicted, in part, by self-efficacy [22]. This would suggest that self-efficacy may be a worthwhile target for cognitive behavioral interventions for LBP.

In addition, there was a significant effect of kinesiophobia in our LBP group; those who had lower TSK scores maintained a longer TTF on the virtual reality Sørensen test; this would suggest that TSK is predictive of performance in individuals with recurrent LBP when provided with a distraction element. On the classic version of the Sørensen test [34], TSK was not predictive of performance in this same group of individuals with recurrent LBP. The virtual reality video game may have actually exacerbated fear cognitions by blocking the participant’s view of the real world, reducing their sense of control, and instead redirecting focus toward their pain-related fear. Moreover, it is possible that attentional resources were reduced in response to the game. Future research could benefit from investigating the response to different types of games to determine whether certain games are more effective or whether games are not effective in any form to counteract pain-related fear.

Previous research has demonstrated an effect of sex on performance on the Sørensen test. Females tend to maintain the test position for longer than males [43,44]; however, several studies have found no sex differences [45,46], and others have found that males maintain the test position for longer than females [47-49]. We did identify a trend toward a sex difference on TTF with females maintaining the position slightly longer than males (*P*=.06). Others have reported that healthy females maintained an isometric trunk extension task markedly longer than healthy males [50]. The authors attributed their results to the muscle mass and strength hypothesis, which describes the relationship among the total muscle mass, vascular compression, and the demand for oxygen. According to this hypothesis, because females typically have lower muscle mass, the vasculature is less compressed during isometric exercise, and the demand for oxygen to the active muscles is lower [51,52]. There is some evidence that females have a greater ratio of type I oxidative muscle fibers in the trunk extensors [53], which would have a greater concentration of beta-2 adrenergic receptors, enhancing vasodilation [54]; there is also evidence that females have a greater degree of capillarization in some muscles [55], enhancing perfusion. However, others have demonstrated that intramuscular pressure may not be associated with a shift in MPF during isometric trunk extension exercises [56,57]. Although muscle mass was not measured in this sample, it is possible that TTF was influenced by perfusion.

### Limitations

As with any study of human subjects, this study is not without its limitations. Our LBP group consisted of individuals with mild, recurrent LBP, which may have also restricted the sample to individuals with low levels of disability and pain-related fear. Individuals with higher disability and pain-related fear have poorer rehabilitation outcomes and typically perform more poorly on the Sørensen test. Thus, significant pain-related fear associations may have emerged in a sample of individuals with more severe kinesiophobia and disability symptoms. In addition, this group was primarily young, fit, college-aged students; future studies will benefit from measuring physical activity levels because cardiorespiratory fitness is likely associated with performance on any endurance task, such as the Sørensen test.

### Conclusions

This study demonstrates that individuals with and without mild, recurrent LBP perform similarly on a variation of the Sørensen test using a virtual reality video game, but the underlying mechanisms driving performance vary between the groups. Performance on this variation of the Sørensen test in healthy individuals is driven primarily by physiological factors, including trunk mass and the MPF slopes of ERS and BF. In addition, trunk mass is an important factor of performance in individuals with a history of recurrent LBP; however, levels of self-efficacy and kinesiophobia also appear to be important predictors of TTF on this virtual reality Sørensen test.

## Abbreviations

BF: biceps femoris
BMI: body mass index
CES-D: Center for Epidemiological Studies-Depression
DOF: degree of freedom
EMG: electromyography
ERS: erector spinae
LBP: low back pain
MPF: median power frequency
PCS: Pain Catastrophizing Scale
PRS: Pain Resilience Scale
TSK: Tampa Scale for Kinesiophobia
TSK-G: Tampa Scale for Kinesiophobia-General Population
TTF: time to task failure
